# Lead and Mercury Exposure and Related Health Problems in Metal Artisan Workplaces and High-Risk Household Contacts in Thimphu, Bhutan

**DOI:** 10.1155/2020/9267181

**Published:** 2020-03-10

**Authors:** Adeep Monger, Karma Wangdi

**Affiliations:** ^1^Royal Centre for Disease Control, Department of Public Health, Ministry of Health, Thimphu, Bhutan; ^2^Occupational Health and Chemical Safety Programme, Public Health Engineering Division, Department of Public Health, Ministry of Health, Thimphu, Bhutan

## Abstract

**Background:**

Metal artisans have been using lead and mercury in their settings for centuries. Exposure to these toxic heavy metals causes adverse health effects. We assessed the occupational exposure of metal artisans and their high-risk household contacts at Thimphu, Bhutan.

**Methods:**

A cross-sectional study in which 134 metal artisan center employees and 48 high-risk household contacts were tested for blood lead and mercury levels. Sociodemographic data, occupational exposure risk factors, and clinical syndrome related to lead and mercury exposure were further obtained and analyzed using EpiInfo 7.0.

**Results:**

The mean age of the metal artisan center employees was 36.02 ± 10.3. The prevalence of elevated blood lead and mercury level was 38.4% and 51.9%, respectively. Significantly higher prevalence of mercury level was observed among the artisans compared to nonartisans (66.97 vs, 16.0). Among three centers, the goldsmith section of the Department of National Properties had the highest (94.1%). Profession as an artisan, mold designing, performing gold amalgamation, working for >8 hours a day, working for >5 years, and working at home were significant risk factors associated with elevated blood mercury level. Significant association was observed between elevated mercury level and complaints of burning or watery eyes (*p*=0.001), anxiety, nervousness, irritability, severe shyness (*p*=0.001), anxiety, nervousness, irritability, severe shyness (*p*=0.001), anxiety, nervousness, irritability, severe shyness (*p*=0.001), anxiety, nervousness, irritability, severe shyness (*p*=0.029), muscle aches (*p*=0.019), and stomach cramps or pain (*p*=0.009).

**Conclusion:**

The prevalence of elevated blood mercury level is concerning among the artisans. Advocacy, proper usage of personal protective equipment, awareness on chemical safety, and hazard associated with lead and mercury usage are needed to minimize the exposure.

## 1. Introduction

Mercury and lead are toxic heavy metals used worldwide causing a huge threat to human and environmental health [1, 2]. The Agency for Toxic Substances and Disease Registry (ATSDR) currently ranks lead and mercury as 2^nd^ and 3^rd^ on their substance priority list based upon the combination of their frequency of toxicity and potential for human exposure [3].

Lead and mercury exposure can occur through the respiratory, gastrointestinal system, and dermal contact [4]. Exposure has toxic effects in almost all organs in the human body as lead and mercury are neurotoxic and cardiotoxic in nature [5, 6]; it affects the gastrointestinal system and causes respiratory illness, hearing loss, and cancer [4, 7]. Lead and mercury exposure is accounted as the most prevalent occupational and environmental health problems globally [2].

Metal artisans are professionals specialized in building traditional ornamental arts and crafts monuments. Lead being highly malleable and ductile, and also due to its density, it is used by the artisans as a weight substitute and to imprint the sculptures into the metals. Mercury is mixed with gold to form an amalgam, which is then applied to metals and sublimed by fire to obtain a rich metallic glow and durable golden appearance. During the subsequent process of fire gold gilding, mercury vapors are released posing occupational and environmental health hazards. Metal artisans are at high risk when they are exposed to lead and mercury via various means such as lead-contaminated dust particles, imprinting and grinding of sculptures, mercury amalgamation process posing a significant risk to their health.

The high-risk household contacts of the artisans are also at the risk of developing lead and mercury toxicity through artisan's contaminated cloths, practicing metal artisan work at home, and living close to the artisan center. Currently, there is a paucity of information on the occupational exposure to lead and Mercury among the artisans and their high-risk household contacts. We, therefore, conducted this study to assess the prevalence of elevated blood level of lead and mercury, associated risk factors, and clinical manifestations in Thimphu, Bhutan.

## 2. Materials and Methods

### 2.1. Study Design and Setting

A cross-sectional observational study was conducted among the employees of metal artisan centers and their high-risk household contacts in Thimphu. Thimphu is the capital city in the western region of Bhutan. Three main artisan centers in the study include Department of National properties (DNP goldsmith and bronze section), Dharma Arts and Crafts Centre (Dharma ACC), and Druk Bronze Casting Centre.

### 2.2. Data Collection and Testing

A standard investigator-administered questionnaire was used to document their sociodemographic characteristics, occupational exposure and safety, and presence of sign and symptoms of lead and mercury exposure. From each participant, 10 ml of venous blood samples were collected in metal-free, evacuated, prescreened, K2 EDTA vacutainer tubes (BD, Franklin Lakes NJ USA). Blood lead and Mercury levels were analyzed using Agilent 7700x inductively coupled plasma mass spectrometry (ICP-MS) as per the method by Ryszard et al. [8].

### 2.3. Data Processing and Analysis

The data were double-entered into Epi-Data Entry version 3.1 (Epi-Data Association, Odense, Denmark) and exported into EpiInfo 7 for analysis. The prevalence of elevated levels of Mercury and lead levels were expressed in the form of percentages. Univariable analysis was performed to examine the unadjusted association of variables with elevated levels of mercury and lead in blood and presented as odds ratio (95% CI). Unpaired two-tailed *t*-test was used to determine the potential differences between the means of selected study groups. A *p* value of <0.05 was considered to be significant.

### 2.4. Ethical Consideration

Ethical clearance was sought from Research Ethics Board of Health, Bhutan, and the study was conducted only after the approval. Informed consent was obtained from all the participants.

## 3. Results

### 3.1. Sociodemographic Characteristics

A total of 177 (134 metal artisan employees and 43 high-risk household contacts) were enrolled in the study. A majority of the study participants was from DNP comprising 58.10%, while Dharma ACC and Druk Bronze Casting Centre employees comprised 32.09% and 9.7%, respectively. The metal artisan employees' age ranged from 17 to 70 years with the mean age of 36.02 ± 10.3 years, and most of the employees were at the age of 25–34 years. Of 134 employees, 109 were artisans and 25 were nonartisan employees comprising office secretariat, night watchmen, and storekeeper (Table 1).

### 3.2. Prevalence

**Figure 1:**
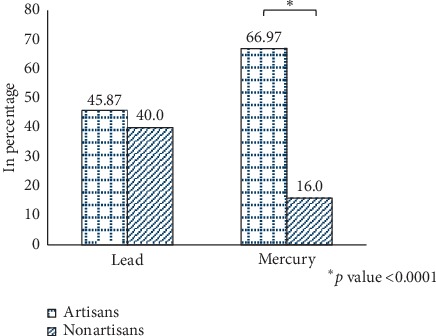
Prevalence of elevated blood lead and Mercury levels among two different occupational groups.

Of the total 177 participants, 68 (38.40%) had elevated blood lead levels (>5 *μ*g/dL interpreted as the abnormal level of lead in blood) and 92 (51.98%) had elevated blood mercury levels (>10 *μ*g/L interpreted as the abnormal level of lead in blood), respectively. Although not much difference in the prevalence of elevated blood lead level was observed between the employees of metal artisan center and high-risk household contacts, there was a significant difference in the blood mercury level (Table 2). Stratifying the occupation of the employees with elevated toxic heavy metal profiles, metal artisans (66.97%) had significantly elevated blood mercury level compared to nonartisans (16.0). There was no notable difference in elevated blood lead level observed between artisans (45.87%) and nonartisans (40.0%).  Figure 1 illustrates the findings.

**Figure 2:**
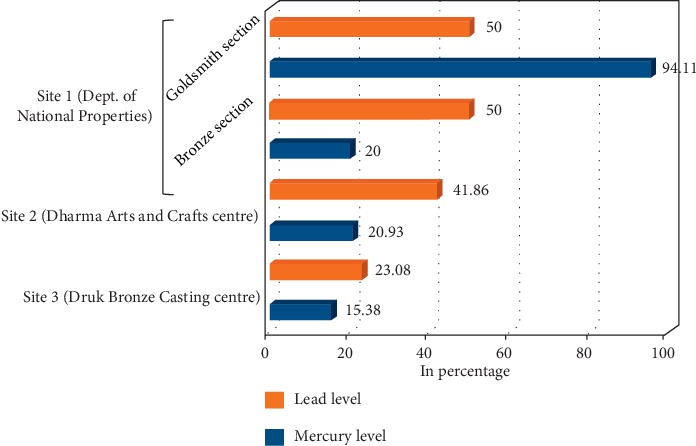
Comparison of prevalence among three sites.

Of the three centers, DNP goldsmith section (94.11%) metal artisan employees had the highest elevated blood mercury level followed by Dharma ACC (20.93%). Both DNP and Dharma ACC had high prevalence of elevated blood lead level among its employees: 50.00% and 41.86% compared to the Druk Bronze Casting Centre (Figure 2).

### 3.3. Risk Factors Associated with Lead and Mercury Exposure

Table 3 shows that working as a metal artisan, working for more than 8 hours in a day, practicing metal artisan work at individual's home, chronically been exposed as a metal artisan for more than five years, mold designing, and performing amalgamation process were significant risk factors for elevated mercury level. The odds ratio of having an elevated blood mercury level was highest practicing a profession as metal artisan (OR 10.64) followed by performing amalgamation process (OR 5.66). There were no significant associated risk factors elucidated with elevation of blood lead level in this study (data not shown).

**Figure 3:**
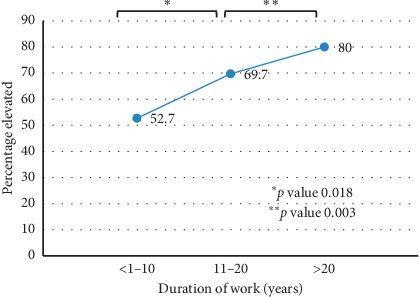
Correlation of elevated blood Mercury and duration of exposure.

**Figure 4:**
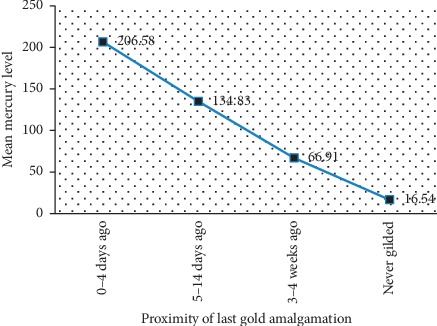
Elevated Hg level due to proximity of last gold amalgamation.

Chronic exposure to mercury is associated with elevation in blood mercury level significantly.  Figure 3 shows that increase in number of years working as metal artisan increases the blood Mercury level in a dose- and time-dependent manner. The amalgamation process being the most significant risk factor was considered further for correlation of elevated mercury level with the proximity of the last amalgamation process.  Figure 4 illustrates that those artisans who performed amalgamation process on a daily basis had 100% elevated blood mercury level with the mean blood mercury level of 206.58 *μ*g/dL compared to those artisans who are involved in amalgamation process but had interruption in between with increased time gap (days and weeks).

### 3.4. Clinical Signs and Symptoms Associated with Elevated Blood Mercury Level

Prominent symptoms among employees of metal artisan centers exposed to mercury included burning or watery eyes (58.4%), headaches (58.4%), anxiety, nervousness, irritability, severe shyness (51.9%), and muscle aches (47.3%). A significant association was found between elevated mercury level in the exposed metal artisans and complaints of burning or watery eyes (*p*=0.001), anxiety, nervousness, irritability, severe shyness (*p*=0.029), muscle aches (*p*=0.019), and stomach cramps or pain (*p*=0.009). Furthermore, above significant clinical manifestations were observed more as their blood mercury level increased in a dose-dependent manner as shown in Table 4.

## 4. Discussion

This study found extremely high blood level of mercury among the artisans, though elevated level of lead was slightly lesser. Metal artisans are exposed to lead and mercury and are at the risk of developing adverse health effects if proper interventions are not initiated.

The prevalence of lead and Mercury level was 44.77% and 57.46%, respectively. This prevalence reported is in comparative with rates ranging from 20.4% to 75.0% for lead [9–12] and 15.0% to 72% elsewhere [11, 13–15]. This possible reason for such variation could be attributed to different setting, cultural context, and environment. The prevalence of elevated blood levels differed significantly between 3 centers, and this is feasibly due to the working environment, number of workers at site, and also the amount of mercury and lead utilized daily. The goldsmith section of DNP uses the most mercury in a daily basis than the other two centers, thus credited to the elevation of mercury level in their settings.

Occupational exposure to mercury and lead via the release of mercury vapors, handling of lead, and the lead dust is a significant concern for the workers. This attributes significantly to health effects of the employees and the environmental health [16]. In the present study, we found the following potential risk factors to be associated with elevated blood levels of mercury: being an artisan, involved in mold designing and amalgamation, working for more than 8 hours/day, working at home, and practicing artisan work for more than 5 years. It is possible that the release of mercury vapor during the amalgamation process may have induced the poisoning. Many studies reported that amalgamation process and duration of work/exposure were significant risk factors for the elevation of blood mercury level [14, 15, 17]. The released mercury vapors possess environmental threat affecting the workers and other population living around them [18]. Similarly, we observed an elevated level of mercury and lead among the high-risk household contacts in our study. Furthermore, we reported for the first time that those artisans who gilded gold during 0–4 days had a mean mercury level of 206.58 *μ*g/L, which decreased with increasing proximity in the gold amalgamation process. The human body has the tendency to excrete these toxic heavy metals in a natural mechanism [19]. Thus, minimizing the time source of exposure has a huge impact in decreasing the mercury burden among the artisans.

The present study observed that neurotoxic symptoms were more prevalent among the artisan that includes anxiety, nervousness, irritability, severe shyness, muscle aches, stomach cramps, forgetfulness, and lack of concentration. Al-Batanony et al. [20] at Quisna Industrial Zone, Egypt, reported the most prominent clinical symptoms as tremors, emotional liability, memory changes, neuromuscular changes, and performance deficits in tests of cognitive function. Studies at Indonesia also recorded that 85% of the gold workers suffered neurological symptoms such as tremors, restricted fields of vision, slow reflexes, sensory disturbances, unbalanced rigidity, and ataxia, thus agreeing in line with our studies [17]. There may be other possible causes for these symptoms, as the study does not have a nonexposed group for comparison. Some of the symptoms may be attributed to aspects of the work environment that are not related to lead or mercury, but which are nevertheless harmful to health. The elevated noise level in the metal artisan center contributes to hearing loss, and possibly headaches. In addition to mercury vapor, smoke from the use of wood or coal heat can cause eye and lung irritation. Muscle aches could be related to the physical demands of the job. Additional data review is necessary to further analyze symptoms that can be specifically attributed to mercury or lead.

This was the first study in the country exploring the prevalence of elevated levels of mercury and lead among the metal artisan workers. This study has its limitations as the similar centers in eastern and central Bhutan were excluded from the study. Therefore, the generalizability of the findings might not be possible.

## 5. Conclusion

The blood levels of mercury and lead among the artisans are elevated. This could possibly be attributed to work environment, lack of awareness on work place safety, and harmful effects of Mercury and lead and on the need to use PPEs. Therefore, advocacy and awareness training programmes on chemical safety and hazard associated with handling of lead and mercury by metal artisans needs to be organized. Responsible administrators need to ensure proper usage of PPEs to minimize the exposure. A monitoring programme and supervision is required to tract the activities of metal artisans so that environment is not contaminated via unscrupulous usage. Furthermore, to reduce the exposure to other high risk household contacts, such as vulnerable population and nearby residents, proper identification of safe designated areas for metal artisan practice and stringent rules for not practicing metal artisan work at home are crucial.

## Figures and Tables

**Table 1:** Sociodemographic characteristics of employees of artisan centers: the participants of the study on blood lead and mercury levels among artisans in Thimphu, Bhutan, 2018.

Sociodemographic characteristics	Total *n* (%)^1^	Male *n* (%)^2^	Female *n* (%)^2^
Employees	134 (100.0)	120 (89.55)	14 (10.45)
High-risk household contacts	43 (100.0)	12 (27.91)	31 (72.09)
Artisan center			
DNP goldsmith section	68 (50.74)	67 (98.55)	1 (1.45)
DNP bronze casting section	10 (7.46)	9 (90.0)	1 (10.0)
Dharma ACC	43 (32.09)	33 (76.74)	10 (23.26)
Druk Bronze Casting	13 (9.7)	11 (84.62)	2 (15.38)
Age (years)			
15–24	15 (11.19)	13 (86.67)	2 (13.33)
25–34	51 (38.06)	40 (78.43)	11 (21.57)
35–44	42 (31.34)	41 (97.62)	1 (2.38)
45–54	20 (14.93)	20 (100.00)	0 (0.00)
55 and above	6 (4.48)	6 (100.00)	0 (0.00)
Literacy (read and write English and Dzongkha)			
Literate	99 (73.88)	86 (86.87)	13 (13.13)
Illiterate	35 (26.12)	34 (97.14)	1 (2.86)
Occupation			
Artisan (metal worker)	109 (81.34)	102 (93.57)	7 (6.42)
Nonartisans^3^	25 (18.65)	18 (72.00)	7 (28.00)

^1^Column percentage, ^2^Row percentage, ^3^Office secretariat, night watchmen, storekeeper.

**Table 2:** Percentage of participants as per the level of blood lead and mercury: study among artisans and their household members in Thimphu, Bhutan, 2018.

Characteristics	Participants *n* (%) with			
	Blood lead		Blood mercury	
	Normal^1^	Elevated^2^	Normal^3^	Elevated^4^
Total (employees of artisan centers + high-risk household contacts) (*n* = 177)	109 (61.5)	68 (38.4)	85 (48.02)	92 (51.98)
Employees of artisan centers (*n* = 134)	74 (55.22)	60 (44.77)	57 (42.54)	77 (57.46)
High risk household contacts (*n* = 43)	35 (81.3)	8 (18.6)	28 (65.12)	15 (34.88)

^1^Blood lead <5 *μ*g/dL, ^2^blood lead >5 *μ*g/dL, ^3^blood mercury <10 *μ*g/dL, and ^4^blood Mercury >10 *μ*g/dL.

**Table 3:** Risk factors associated with elevated blood Mercury level.

Risk factors		Elevated Hg level^*∗*^		OR	95% CI	*p* value
		Yes	No			
Artisan	Yes	73	36	10.64	3.40–33.33	0.003
	No	4	21			
Mold designing	Yes	35	9	3.3	1.39–7.83	0.005
	No	40	34			
Amalgamation process	Yes	45	9	5.66	2.37–13.49	0.001
	No	30	34			
Work hour >8 hrs.	Yes	37	13	3.13	1.45–6.71	0.002
	No	40	44			
Working at home	Yes	43	23	1.51	0.7–313.77	0.002
	No	33	27			
Working as artisan for >5 years	Yes	67	39	3.09	1.29–7.36	0.008
	No	10	18			

^*∗*^>10 *μ*g/L.

**Table 4:** Percentage of participants who reported having clinical symptoms related to mercury exposure within the past 6 months of the study on blood mercury level among employees at artisan worksites in Thimphu Bhutan, 2018 (*n* = 131).

Clinical symptoms^1^	Participants reporting clinical symptoms occasionally or often by blood mercury level *n* (%)				
	All blood Hg levels (*n* = 131)	Blood Hg ≤ 10 (*n* = 55)	Blood Hg > 10 to ≤100 (*n* = 35)	Blood Hg > 100 (*n* = 41)	*p* value^3^
Burning or watery eyes	76 (58.46)^2^	25 (46.30)^2^	21 **(60.00)**	30 **(73.17)**	0.001
Headaches	76 (58.46)^2^	27 (50.00)^2^	21 **(60.00)**	28 **(68.29)**	0.081
Anxiety, nervousness, irritability, severe shyness	68 (51.91)	25 (45.45)	18 **(51.43)**	25 **(60.98)**	0.029
Muscle aches	62 (47.33)	22 (40.00)	15 **(42.86)**	25 **(60.98)**	0.019
Stomach cramps or pain	57 (43.51)	20 (36.36)	17 **(48.57)**	20 **(48.78)**	0.009
Increased tiredness	50 (38.17)	13 (23.64)	17 **(48.57)**	20 **(48.78)**	0.162
Forgetfulness, lack of concentration	54 (41.22)	26 (47.27)	11 (31.43)	17 (41.46)	0.602
Metal taste in mouth	27 (20.61)	11 (20.00)	6 (17.14)	10 (24.39)	0.725
Decreased hearing	28 (21.54)^2^	12 (22.22)^2^	7 (20.00)	9 (21.95)	0.928
Clumsiness or tremors	15 (11.45)	5 (9.09)	5 (14.29)	5 (12.20)	0.792
Vomiting, diarrhea, or constipation	23 (17.56)	13 (23.64)	6 (17.14)	4 (9.76)	0.211
Difficult breathing, shortness of breath	18 (13.74)	10 (18.18)	5 (14.29)	3 (7.32)	0.325
Skin rash	16 (12.21)	7 (12.73)	6 (17.14)	3 (7.32)	0.577
Increase in salivation (drooling)	12 (9.23)^2^	6 (10.91)	4 (11.43)	2 (5.00)^2^	0.649

^1^Self-reported by the participants, ^2^missing = 1, and ^3^comparison between blood Hg ≤ 10 and > 100 *µ*g/dL.

## Data Availability

The data used to support the finding of this study are included within the article.

## References

[1] World Health Organization (2018). Exposure to mercury: a major public health Concern. https://www.who.int/ipcs/features/mercury.pdf.

[2] World Health Organization (2018). The public health impact of chemicals: knowns and unknowns. https://www.who.int/ipcs/publications/chemicals-public-health-impact/en/.

[3] Agency for Toxic Substance and Disease Registry (2018). The ATSDR 2017 substance priority list. https://www.atsdr.cdc.gov/spl/.

[4] Jaishankar M., Tseten T., Anbalagan N., Mathew B. B., Beeregowda K. N. (2014). Toxicity, mechanism and health effects of some heavy metals. *Interdisciplinary Toxicology*.

[5] Levine S. P., Cavender G. D., Langolf G. D., Albers J. W. (1982). Elemental mercury exposure: peripheral neurotoxicity. *Occupational and Environmental Medicine*.

[6] Fernandes A. B., Barros L. F., Pecanha F. M. (2012). Toxic effects of mercury on the cardiovascular and central nervous systems. *Journal of Biomedicine and Biotechnology*.

[7] Liao L. M., Friesen M. C., Xiang Y.-B. (2016). Occupational lead exposure and associations with selected cancers: the Shanghai men’s and women’s health study cohorts. *Environmental Health Perspectives*.

[8] Gajek R., Barley F., She J. (2013). Determination of essential and toxic metals in blood by ICP-MS with calibration in synthetic matrix. *Analytical Methods*.

[9] Weems K., Bostwick J., Shen T. (2015). *Trends in Elevated Blood Lead Levels in Adults- Illinois, 2005–2014*.

[10] Alarcon W. A. (2016). Elevated blood lead levels among employed adults-United States, 1994–2013. *MMWR Morbidity Mortaityl Weekly Report*.

[11] Osorio García S. D., Hernández Flores L. J., Sarmiento R. (2014). Prevalencia de mercurio y plomo en población general de Bogotá 2012/2013. *Revista de Salud Pública*.

[12] Saliu A., Adebayo O., Kofoworola O., Babatunde O., Ismail A. (2015). Comparative assessment of blood lead levels of automobile technicians in organised and roadside garages in Lagos, Nigeria. *Journal of Environmental and Public Health*.

[13] Steckling N., Bose-O’Reilly S., Pinheiro P. (2014). The burden of chronic mercury intoxication in artisanal small-scale gold mining in Zimbabwe: data availability and preliminary estimates. *Environmental Health*.

[14] Steckling N., Tobollik M., Plass D. (2017). Global burden of disease of mercury used in artisanal small-scale gold mining. *Annals of Global Health*.

[15] Diaz S., Munoz-Guerrerro M. N., Palma-Parra M. (2018). Exposure to mercury in workers and the population surrounding gold mining areas in the Mojana region, Colombia. *International Journal of Environmental Research and Public Health*.

[16] Roberts H. W., Charlton D. G. (2009). The release of mercury from amalgam restorations and its health effects: a review. *Operative Dentistry*.

[17] Abbas H. H., Sakakibara M., Sera K. (2017). Mercury exposure and health problems in urban artisanal gold mining (UAGM) in Makassar, south Sulawesi, Indonesia. *Geosciences*.

[18] Gibb H., O’Leary K. G. (2014). Mercury exposure and health impacts among individuals in the artisanal and small-scale gold mining community: a comprehensive review. *Environmental Health Perspectives*.

[19] Ye B. J., Kim B. G., Jeon M. J. (2016). Evaluation of mercury exposure level, clinical diagnosis and treatment for mercury intoxication. *Annals of Occupational and Environmental Medicine*.

[20] Al-Batanony M. A., Abdel-Rasul G. M., Abu-Salem M. A. (2013). Occupational exposure to mercury among workers in a fluorescent lamp factory, Quisna industrial zone, Egypt. *International Journal of Occupational and Environmental Medicine*.

